# The Mental Health Impacts of Internet Scams

**DOI:** 10.3390/ijerph22060938

**Published:** 2025-06-14

**Authors:** Luke Balcombe

**Affiliations:** Australian Institute for Suicide Research and Prevention, School of Applied Psychology, Griffith University, Messines Ridge Road, Mount Gravatt, QLD 4122, Australia; lukebalcombe@gmail.com

**Keywords:** scams, internet, mental health impacts, victim insight, artificial intelligence

## Abstract

Internet scams have become more sophisticated and prevalent in countries such as Canada, the US, the UK, and Australia. Australia has made some progress in effective scam intervention strategies and seen possible growth in public awareness. However, there is a lack of insight into factors associated with profound shame and embarrassment, emotional distress such as anxiety and depression, and trauma and suicide in scam victims. To fill this gap, this perspective paper aimed to provide insight into the factors associated with the negative mental health impacts of internet scams by integrating a narrative literature review with a victim case study detailing a group’s experience of an investment scam in Australia. It found that internet scams cause emotional and social issues like depression, anxiety, trauma, and isolation, mostly prolonged upon substantial loss. The author’s insight into the intensely negative mental health impacts of an investment scam allows for the presentation of a group who struggled to access adequate support and mental health care in their response to insidious organized crime. Better education, resilience-building, and support systems are needed. These shortcomings call for strategies for tailored digital mental health services such as emotionally attuned, trauma-informed digital companionship through human-like artificial intelligence (AI) applications.

## 1. Introduction

Scams are a common and sophisticated issue in today’s digital age, with scenarios involving the internet, as well as telephone calls and text messages [1,2,3]. The damage to financial well-being in scam victims has been examined more than the profound psychological impact [4,5,6,7]. The global increase in internet scams since the COVID-19 pandemic led to a call for mental health professionals to provide trusted sources of education on cybersecurity [8]. While Canada, the US, and the UK have seen rising scam losses, Australia saw a significant drop in average losses in early 2025 compared to previous quarters, indicating effective intervention strategies and possible growth in public awareness [9]. The need for insight into factors associated with individuals’ experiences called for research into the profound and hidden mental health impacts of internet scams manifesting as emotional distress, including depression, anxiety, shame, and embarrassment [10].

The aim of this perspective is to provide insight into current findings on internet scams by reviewing the literature and media articles and comparing them with an intrinsic case study of individuals’ negative mental health impacts from an investment scam in Australia. Additionally, it discusses the potential of tailored digital mental health services to effectively deploy human-like artificial intelligence (AI) applications to increase moderation, accessibility, and help-seeking as well as reduce emotional distress and trauma in scam victims.

## 2. Methodology

This perspective integrates (1) a narrative literature review (i.e., purposive sample of media articles and journal articles) with (2) a case study involving the author embedded in a scam victim group.

The narrative literature review is adapted from the four steps outlined by Demiris et al. [11]: (1) conduct a search of numerous databases and search engines; (2) identify and use pertinent keywords from relevant articles; (3) review the abstracts and text of relevant articles and include those that address the research aim; and (4) document results by summarizing and synthesizing the findings and integrating them into the review.

A preliminary online search found that there are not enough studies that meet the strict criteria of a systematic review. Therefore, a purposive sample of articles was applied to demonstrate an educational approach to the novel topic. By selectively choosing relevant articles and reviewing them thoroughly, valuable insights were compiled into a coherent narrative literature review. This method enabled flexibility in considering diverse perspectives, providing a more comprehensive understanding of the topic.

Peer-reviewed journal articles and media articles were obtained through systematic searches of computerized databases and targeted online searches. The databases searched were Scopus, ScienceDirect, Sage, and the Association for Computing Machinery (ACM) Digital Library. The search engines used were PubMed, Google Scholar, and IEEE Xplore. The search terms used were “internet scams” OR “cybercrime OR “investment scams” AND “mental health impact” OR “psychological impact”.

The following selection criteria were used:

Inclusion criteria:

- Studies that have been published in peer-reviewed journals and media articles;
- Studies that have been published in the English language;
- Studies that have been published between 2010 and 2025;
- Studies that have investigated the mental health impact of internet scams;
- Studies that have reported on internet scams, how they operate, current issues, and their financial and mental health impact on victims.

Exclusion criteria:

- Studies that have not been published in peer-reviewed journals and media articles;
- Studies that have not been published in the English language;
- Studies that were published before 2010;
- Studies that have investigated the mental health impact of internet scams;
- Studies that have not reported on internet scams, how they operate, current issues, and their financial and mental health impact on victims.

Relevant articles and their reference lists were explored based on (1) relevance to the guiding research aim (2) the inclusion of examples of theoretical and empirical research and (3) the highlighting of issues and possible solutions. These articles were used for a thorough and unbiased analysis of current knowledge, utilizing a systematic approach to minimize bias through comprehensive searches and relevant article focus. Despite this, bias in the literature was critically assessed, and any limitations were noted.

This perspective also applies an intrinsic case study approach as designed by Stake because it is inherently interesting [12]. It focuses on qualitative inquiry and interpretive analysis within a real-world context to understand unique perspectives in a specific investment scam of which the researcher was also a victim [12]. Five steps—case selection, data collection, data analysis, narrative development, and reflexivity with ethical considerations—guide the detailed exploration and presentation of a unique case study.

The lead author led the case study with member checks by a fellow scam victim to delve deeply into individual and collective experiences of an investment scam in Australia. The case study started with archival research; the author examined historical records, including media articles, witness statements, and conversations with police detectives and significant others (e.g., investigators and informants), and examined financial/legal documents, to trace the evolution of scam tactics as well as financial/mental health impacts on victims.

Data collection involved a questionnaire with scam victims (*n* = 25) and detailed interviews (*n* = 3) to capture the financial and emotional toll of the investment scam. The case study was structured around adult Australian resident scam victims. The group’s interactions with the scam were scrutinized, revealing patterns in mental health impacts, vulnerability, and trust dynamics, as well as coping mechanisms. This included personal narratives to paint a comprehensive picture of the phenomenon.

The questionnaire was collated through email (addresses of scam victims were supplied by a police detective). The questions asked about the financial and mental health impacts of the scam experienced by individuals. In-depth interviews via phone calls with scam victims helped identify emotional triggers and psychological consequences. Thematic coding was employed to uncover recurring themes in the investment scam narratives. Since the lead author was involved as a scam victim and researcher, participants were informed that their participation was voluntary and on a victim-to-victim/researcher basis. Contact details for mental health support and crisis services were supplied to participants.

## 3. Literature Review Findings

### 3.1. The Global Epidemic of Internet Scams

Scams, involving deceptive practices to defraud individuals, cost victims over USD 1 trillion in 2023 [13]. The Global State of Scams Report in 2022 revealed that only 7% of scams are reported, highlighting the issue’s vast scale [14]. Internet scams have become a global epidemic, with the digital economy’s growth making scams the most reported crime in many countries, including developing nations where mobile scams are rampant. Investment scams, especially those involving imposters using the internet, money mule accounts, and cryptocurrencies have surged due to economic pressures, causing significant losses [15,16,17,18,19,20,21]. The rise in scams reflects the dark side of a digitalized world economy during global crises [22], weakening trust in digital platforms due to cybersecurity threats from malicious internet provider (IP) addresses and unsafe browsing [23].

### 3.2. The Growing Sophistication of Internet Scams

Scammers use tactics like impersonating banks, businesses, and authorities, phishing for personal information, setting up fake websites, offering deceptive investment schemes, creating urgency, and false lottery notifications, as well as tech support and romance scams [24,25].

People fall for scams due to financial desperation, promises of quick wealth, social engineering, false legitimacy, a lack of awareness about sophisticated scam tactics, emotional triggers, exploited trust, and busy lifestyles leading to missed red flags and insufficient verification [26,27,28]. Scams have become more sophisticated with AI for voice-cloning, deepfakes, chatbots [29,30], phishing schemes [31,32,33], data tracking/sharing, targeted ads, and cloned investments [34,35,36,37]. Psychological tactics include impersonation and fake ‘social proof,’ as well as deceptive authorized push payments and payment redirections [38,39,40,41,42,43]. There is also a lack of awareness about technological solutions like server-side tracking, Virtual Private Networks (VPNs), and AI chatbots to counter these issues [44,45,46,47,48].

Big tech companies play a crucial role in preventing scams and raising awareness [49]. However, United States (US) laws’ opacity has hindered an understanding of how these companies profit from scam ads on their platforms [50]. Collaboration among US government agencies, law enforcement, financial institutions, telecommunications companies (telcos), consumer organizations, the private sector, and individuals is essential for combating scams [48,49,50,51,52]. In Australia, a federal scam protection campaign aims to fine tech giants, banks, and telcos penalties up to AUD 50 million and compensate victims, differing from the UK model, which forces banks to reimburse customers unless they acted fraudulently or with gross negligence [52,53,54,55,56,57]. The proposed Australian framework introduces obligations for companies to detect, report, disrupt, and respond to scams.

### 3.3. The Targeting of Internet Scam Victims

Internet scams can affect anyone, anywhere [58,59,60]. The warnings of a real risk to older people (specifically for financial cybercrime) [61] came before findings that they are less vulnerable to being scammed online than in general [62]. A systematic review found that older adults are susceptible to being online fraud victims [63]. While there is less prevalence than other age groups, they are vulnerable because of their cognition, trust, and reduced internet experience and awareness of how the internet is used to deceive them.

Media reports show how the internet enables scams. For example, foreign call center workers target Americans and Australians with investment scams from South-East Asia, the Middle East, and Eastern Europe [64,65]. One in four Australian teenagers fall victim to social media scams, including sextortion, fake websites, and illegitimate online marketplace trading [66]. Constant cyber-attacks on Australian banks and scams by overseas actors necessitate practical steps for financial services and customers to counter these risks [67,68,69].

Experts share tips on scam protection [70,71], aligning with police efforts on scam detection and disruption. However, scammers adapt their tactics based on available feedback, exploiting psychological vulnerabilities, cognitive biases, and overconfidence through manipulative communication, urgency, and rapport-building [72,73].

### 3.4. The Literature on the Psychological Impacts of Scams

The psychological impacts on scam victims is severe, leading to distress, anxiety, depression, post-traumatic stress disorder (PTSD), and suicidality [74,75,76,77]. Victims often feel shame, guilt, anger, helplessness, and fear as they deal with the financial and emotional fallout [78]. Awareness and vigilance are crucial in preventing scams [79,80], helping victims avoid self-blame, encouraging reporting, and learning from experiences to prevent future scams. There is a need for greater use of psychological approaches in the prevention of cybercrime as well as empowering resilience against scams [81].

The literature to date includes understanding how scams work from a financial crime or technology perspective. The focus has been on detecting and analyzing scam sites [82], while other studies have shown scammer or victim behavior before or during the fraud [83,84,85,86]. However, a systematic review of the psychology of internet fraud victimization showed susceptibility in those who respond to time-limited communications [27]. There was a call for investigating how mood and emotion affect engagement with internet scams.

Romance scams are the most studied of the internet scam genre. However, recent reviews indicate that online romance scams are under-researched despite a rise in studies [87,88]. The persistent low reporting rates of romance scams is due to embarrassment or fear of disapproval. The isolating experience of being a romance scam victim can lead to individuals finding ways to cope without understanding from family and friends [89]. Such victims tend to be middle-aged, well-educated women who are impulsive, less kind, and more trustworthy and have an addictive disposition [90]. They feel marginalized by victimization and experience persistent deep emotional shifts hindering their ability to self-regulate potential suicidality [91].

A cross-sectional study on internet scams found negative mental health impacts and that individuals with poor mental health may be more susceptible to becoming victims [92]. However, no causal relationship was established, and it is unknown for how long the impacts of an internet scam experience on mental health may last. Financial losses from internet scams may worsen mental health issues or trigger new ones. The limited findings on internet scams require developing an evidence base from narration.

### 3.5. Case Study of an Internet Scam

In 2023–2024, a group of Australian investors (*n* = 25) fell victim to an elaborate internet scam. The investment scam, operated by a global syndicate, preyed on internet users who conducted an online search for term deposits or bonds. This digital footprint led to fake websites on Google or Facebook advertisements. The victims’ contact details were provided to ‘investment advisors’ who subsequently contacted them by phone and email, impersonating a legitimate business with an Australian financial services license. The scam involved developing rapport over time, authentic-looking investment guides, online portals, and payment instructions to an ‘escrow account’ used by money mules to forward money to other individuals and companies as well as currency exchange and cryptocurrency platforms.

The unique perspective brought by the author being part of the scam victim group became clear after a police detective tracked down 25 victims throughout Australia. This detective connected them in 2024–2025 with emails and phone calls, and assisted them in fighting back and reaching out to share personal insights into the financial and mental health impacts.

Some victims lost substantial sums, and others lost more modest sums, while a few avoided substantial financial losses after canceling the payment and/or successful bank recalls. A few months after the group connected, some victims (including the author) thereafter followed up with emails and phone calls to collate a summary of the financial aspects of the scam. The dissatisfaction with the banks and the Australian Financial Complaints Authority (AFCA)’s handling of investigations/complaints was the impetus for a spreadsheet summary. It was overall critical of the response of banks and AFCA, highlighting a perceived lack of genuine consideration, assistance, accountability, or empathy from these institutions.

In terms of the mental health impacts of the scam, the group reported significant psychological distress manifesting as insomnia, anxiety, depression, and trauma (notably, post-traumatic stress disorder). Insomnia was noted as temporary while awaiting the decision of bank recalls (1–2 months). The experience of anxiety and depression was prolonged in those who suffered major financial loss. It was compounded by a perception of not being adequately served by banks and AFCA as well as unproductive and/or slow police inquiries (up to 2 years if investigated). Those who were satisfied with bank recalls had temporary anxiety and depression (1–2 months). PTSD was commonly still present long after the scam (1–2 years), regardless of whether the financial loss was significant.

In the months after the victims learned about the scam, there were reports of persistent sadness, loss of interest, feelings of panic, and excessive worry, typical symptoms of depression and anxiety. Some scam victims were taking anti-depressive or other psychotropic medications to help them cope 1–2 years after a substantial loss. As a result of the shame of having been scammed, some withdrew from social interactions, felt isolated, and experienced loneliness and despair as well as a relationship breakup. This occurred up to 1–2 years after the scam for some victims. Constant rumination over the incident exacerbated these feelings, complicating daily life. Financial security and personal information became sources of heightened worry and anxiety. Some of the group consulted their doctor to access mental health care in the first months of realizing they were scammed, while most did not. A few found solace in sharing their experience with fellow victims, and some of this sharing occurred 1–2 years after being scammed. Overall, there were various negative mental health impacts and emotions; for some victims there were short-lived effects, while for others they were long-term.

## 4. Discussion

### 4.1. The Fight Against Internet Scams

Research and media reports highlight the widespread impact of internet scams, exploiting global loopholes such as through search engines and social media. Vulnerable groups include older adults, due to trust and limited digital literacy, and teenagers, targeted on social platforms. Scammers cause significant financial and psychological harm, underscoring the need for systemic reforms.

Australia provided a timely perspective as financial impacts from scams showed signs of decline for the first time since 2015, as noted in AFCA’s 2023–2024 annual review [93]. In September 2024, the Australian Government’s Treasury introduced anti-scam codes mandating businesses to implement strong measures, enforceable with penalties up to AUD 50 million, and victim compensation provisions if the business fails to meet the standards [94]. These efforts align with the creation of Australia’s National Anti-Scam Centre (NASC).

Australia’s recent anti-scam measures show progress in detecting and disrupting scams, yet the response to victims’ financial and mental health impacts remains inadequate. The case study highlights dissatisfaction with the care from banks and financial complaints groups, along with gaps in educational resources for recovery and suicide prevention. Media reports and the literature emphasize the urgent need for stronger victim support, empowerment, and tools to rebuild trust, as victims often face isolation and emotional distress. While some efforts exist to combat scams, resilience and faster preventive measures are still under-explored.

### 4.2. Comparison of Findings on the Mental Health Impacts of Internet Scams

An online survey conducted in 2025 sought insight into the profound and hidden mental health impacts of internet scams, showing that victims suffer from depression, anxiety, shame, and embarrassment. This perspective also aimed to provide insight into the negative mental health impacts by comparing literature review and case study findings to address the lack of research on these effects.

Literature Review Findings:

- Scam victims often experience severe emotional distress, including depression, anxiety, shame, embarrassment, and PTSD.
- Romance scams are highlighted as particularly emotionally damaging, with victims reporting feelings of marginalization and deep emotional shifts that hinder self-regulation, occasionally leading to suicidality.
- Victims frequently deal with persistent sadness, anger, guilt, fear, and helplessness, which exacerbate their psychological struggles.
- Despite the significant psychological toll, scams are under-studied in terms of their mental health impacts compared to financial losses.
- There is limited exploration of resilience and coping mechanisms among scam victims, as well as insufficient educational resources for mental health recovery and suicide prevention.

Case Study Findings:

- Victims of the investment scam in Australia reported psychological distress manifesting as temporary insomnia, ongoing anxiety and depression (associated with substantial financial loss), and prolonged trauma, notably PTSD.
- Symptoms typically present over months included persistent sadness, loss of interest in activities, feelings of panic, excessive worry, and rumination over the incident.
- Social withdrawal, isolation, loneliness, and relationship breakups were common among victims for long periods due to the shame associated with being scammed.
- Heightened anxiety surrounding financial security and personal information became prominent after the scam.
- Though some victims sought medical help or mental health care, most did not, highlighting barriers to accessing support and recovery resources.

Comparison of Findings:

- Both the literature review findings and case study emphasize the significant emotional distress caused by internet scams, including persistent depression, anxiety, shame, and PTSD (most prolonged).
- The literature review provides broader insight into psychological impacts across different types of internet scams globally, while the case study offers a focused perspective on the mental health toll of a specific investment scam in Australia.
- Victims in the case study reported similar symptoms to those outlined in the literature, but their experiences also revealed additional social and relational impacts, such as withdrawal and relationship breakups.
- The literature review highlights the need for resilience-building and educational resources, while the case study underscores the inadequacies of current support systems and the isolation felt by victims.

### 4.3. Digital Mental Health and AI Solutions for Internet Scam Responses

Digital mental health (DMH) services complement traditional support [95], empowering internet scam victims to overcome shame and embarrassment in private with practical strategies for emotional regulation and recovery. Although digital mental health services may connect victims to traditional support, conventional methods often fail to meet their needs effectively. For example, there is a need for new approaches to move beyond the limited effectiveness of psychotropic drugs [96] and fill the gap left by the scarcity of mental health professionals [97,98].

A help-seeking solution is to use AI-driven mental health applications, such as Wysa, Youper, and Woebot [99,100,101]. AI chatbots offer (cost)-effective treatment mainly for anxiety and/or depression [102], usually with positive engagement outcomes [103].

Conversational AI, such as AI chatbots, offers accessible and convenient mental health care, allowing individuals to receive therapy regardless of location, cost, stigma, or schedule [104,105]. The availability of AI can help overcome the persistent low reporting rates for scams. While available 24/7, DMH services lack the empathetic human touch of in-person therapy and face challenges like privacy concerns, data security, and algorithmic biases, along with limitations in addressing complex emotions. The randomized controlled trial (RCT) for Therabot—a generative AI therapy chatbot—shows promising preliminary effectiveness [106].

More research and development are required to increase the safety, usability, and effectiveness of AI chatbots [104]. While human intervention may be necessary in crisis situations, there is also the issue of AI chatbot hallucinations, and the need to address biases or errors. For example, AI chatbots can be a problem if the app crashes and a conversation is lost. Memory-enabled sessions provide personalized interactions, while clean sessions treat each as new. Combining both ensures efficiency and avoids biases, offering flexibility to users.

AI integration in scam prevention includes fraud protection in banks and monitoring online platforms to flag suspicious activities, reducing victimization and psychological harm. AI can also educate individuals on scam tactics and provide real-time alerts. However, tailored digital mental health services like lived experience-guided, emotionally attuned, trauma-informed AI companions are lacking [107].

Human-like AI chatbots with emotional intelligence, informed by lived experiences, could address dissatisfaction with systemic support by offering ethical, culturally sensitive assistance. These applications should prioritize co-regulation to help scam victims manage trauma and emotional distress effectively.

## 5. Conclusions

Scams have significant negative mental health impacts, leading to stress, anxiety, depression, and isolation. In the months after, victims often experience shame and embarrassment, exacerbated by financial insecurity and a lack of support. Severe cases may even trigger suicidal thoughts. Recognizing these impacts is essential for providing effective preventive measures and recovery support, some of which may be required years later.

A case study on an investment scam in Australia revealed victims’ struggles with temporary insomnia and ongoing trauma, anxiety and depression, as well as social withdrawal. While some found solace in shared experiences, dissatisfaction with systemic responses was common. The study underscores the importance of innovative solutions like human-like AI chatbots, which could offer scalable, accessible, and empathetic emotional support.

Internet scams highlight the need for resilience-building resources, improved educational outreach, and integrated mental health systems. By leveraging advancements such as AI-driven emotional intelligence tools, recovery pathways can be enhanced, bridging gaps left by traditional systems and addressing the profound emotional distress associated with scams.

Finally, there is a need for strategies aimed at combining prevention and intervention with victim support, emphasizing the importance of education and technological interventions. In sum, this perspective has shed light on the intricate ways in which scams operate and affect individuals, paving the way for more targeted preventive measures and support systems.

## Data Availability

No new data were created or analyzed in this study. Data sharing is not applicable to this article.

## Abbreviations

ACM	Association for Computing Machinery
AFCA	Australian Financial Complaints Authority
AI	Artificial intelligence
DMH	Digital mental health
IEEE	The Institute of Electrical and Electronics Engineers
IP	Internet provider
NASC	National Anti-Scam Centre
RCT	Randomized controlled trial
PTSD	Post-traumatic stress disorder
UK	United Kingdom
US	United States
VPNs	Virtual private networks
